# Spatial selectivity in visual detection suffers when attention is divided

**DOI:** 10.3758/s13414-025-03066-x

**Published:** 2025-04-11

**Authors:** John Palmer, Cathleen M. Moore, Alex L. White, Geoffrey M. Boynton

**Affiliations:** 1 Department of Psychology, University of Washington, Seattle, WA 98195 USA; 2 Department of Psychological and Brain Sciences, University of Iowa, Iowa City, IA 52242 USA; 3 Department of Neuroscience and Behavior, Barnard College, New York, NY 10027 USA

**Keywords:** Selective attention, Spatial filtering, Selection, Divided attention

## Abstract

Humans are not perfect at selectively responding to one stimulus while ignoring others visible at the same time. In *spatial filtering tasks*, this imperfect selectivity is often measured by how the judgment of the relevant stimulus depends on whether an irrelevant stimulus is associated with the same response. Such *congruency effects* decline with increasing spatial separation between stimuli and are minimal for widely separated stimuli. However, there is evidence that divided attention can increase congruency effects even for widely separated stimuli. We investigated this possibility for a pair of widely separated stimuli and a simple yes/no detection task. Performance was measured for a single task (only one of the stimuli was task-relevant) and for a dual task (both of the stimuli were task-relevant). In the single task there were small congruency effects, whereas in the dual task larger congruency effects occurred despite the widely separated stimuli. Results from a second experiment with sequential and simultaneous presentations were consistent with the congruency effect being due to later processes such as memory or decision rather than immediate processes such as perception. Additional results comparing high and low performance levels were consistent with congruency effects being due to a graded process such as attenuation or crosstalk rather than an all-or-none process such as blocking or substitution. These results rule out many possible theories of spatial selectivity. Our working hypothesis is that spatial selection can protect against interactive processing of multiple stimuli for a single task but not for dual tasks.

## Introduction

Because the brain encodes many stimuli simultaneously, it often has multiple conflicting sources of information. The brain must therefore be able to attend selectively to stimuli at relevant locations without being influenced by stimuli at irrelevant locations. For example, when driving to a pharmacy that you know has a blue sign and is on the right side of the road, attending to the right side helps you avoid accidentally turning towards similarly colored signs that are on the left. But even in simple laboratory tasks, such spatial selectivity is imperfect: in many cases, an observer's response to a stimulus at one location is influenced by the stimuli at other locations. In this study, we investigate how spatial selectivity depends on the number of simultaneous task-relevant locations.

One way to measure spatial selectivity is by congruency effects in a spatial filtering paradigm*.* When observers are simultaneously presented with a relevant stimulus and an irrelevant stimulus, congruency effects are the difference in performance between trials in which the stimuli are associated with the same response and trials in which the stimuli are associated with different responses. Such congruency effects are common in studies of the limits of selective attention using spatial filtering (e.g., Eriksen & Hoffman, 1973; Yantis & Johnston, 1990). Consider the early example of congruency effects in spatial filtering by Eriksen and Hoffman (1973). They presented a circular display of letters from the set {A, U} or from the set {H, M}. They cued one location and asked observers to categorize the letter at that cued location into one of the two possible sets while ignoring letters at other locations. They analyzed performance as a function of whether nearby letters were from the same set as the target *(congruent)* or from the other set *(incongruent)*. Responses to targets with congruent neighbors were faster than responses to targets with incongruent neighbors. Moreover, responses to trials with incongruent neighbors that were immediately adjacent to targets were slower than those on which the incongruent neighbors were further away. This modulation reveals spatial selectivity.

In this article, we investigated how the spatial selectivity of spatial filtering is affected by divided attention. Specifically, consider a dual task in which two stimuli are presented and the participant is instructed to judge them independently with two separate responses. Is spatial filtering less effective when both stimuli must be processed even though only one stimulus is relevant to each response? Moreover, how do such dual tasks compare to when one stimulus is relevant and the other stimulus is entirely irrelevant (i.e., single tasks). There are results in the literature showing that congruency effects increase for dual tasks compared to single tasks (e.g., Bonnel et al., 1992; Logan & Gordon, 2001). This is inconsistent with typical theories of spatial selectivity that assume local perceptual processes are independent for widely separated stimuli. It is also inconsistent with typical theories of divided attention that pose limits on processing capacity and not selectivity. To address these unexpected results, we examine the properties of congruency effects for single and dual tasks. The results help discriminate between alternative theories of spatial selection and of divided attention.

Before proceeding, it is helpful to elaborate how spatial filtering is distinct from other paradigms used to study spatial selective attention and why it is the focus of the current study. First compare spatial filtering to partially-valid cueing (Posner, 1980). In spatial filtering, some stimuli are relevant (*targets*) and must be responded to because they appear in a cued location, whereas other stimuli are irrelevant (*foils*) and must not be responded to because they appear in an uncued location. In partially-valid cueing, by contrast, there are no irrelevant stimuli. Instead, the probability of where a relevant stimulus is likely to occur is varied and cued. Given these differences, partially-valid cueing is useful for studying the differential allocation of attention among multiple spatial locations in which relevant stimuli can appear, whereas spatial filtering is useful for studying spatial selection of relevant stimuli to the exclusion of irrelevant stimuli. A direct comparison of these paradigms was conducted in Yigit-Elliot et al. (2011). Second, compare spatial filtering to the flanker paradigm (Eriksen & Eriksen, 1974; Eriksen & Schultz, 1979). Again, in spatial filtering, a relevant stimulus is specified by only whether it is in a cued location or not, and therefore, the task depends on spatial selection. In the flanker paradigm, by contrast, the relevant stimulus is specified by multiple cues, designed to maximize the successful selection of the target stimulus. Cues in the flanker paradigm include spatial location, typically combined with foveal positioning, the relative position within a multiple stimulus array (typically the center), and sometimes other stimulus properties such as color (Harms & Bundesen, 1983). Given these differences, spatial filtering is useful for studying the properties of spatial selectivity which is the focus of the current study, whereas the flanker paradigm is useful for revealing processing interactions that occur despite excellent cues for selection (e.g., crosstalk; Navon & Miller, 1987). In summary, spatial filtering is specialized to reveal spatial selectivity between relevant and irrelevant stimuli.

### Studies of spatial selectivity using the spatial filtering paradigm

To quantify spatial selectivity, our lab has conducted several studies of spatial filtering using two disks in the periphery (see Palmer & Moore, 2009, for a review of other approaches). One peripheral location is cued as relevant and then two disks are briefly displayed with one at the relevant location and another at an irrelevant location with the same eccentricity. The observer must make a judgment about the relevant disk and ignore the irrelevant disk. In the most relevant of these studies for current purposes (Yigit-Elliot, 2012), each disk had a color that was chosen from one of two possible categories such as {“red,” “green”} versus {“blue,” “yellow”} and the task was to judge the color category of the relevant disk. The colors for the relevant and irrelevant disks were independent and thus half the time they were from the same category (*congruent*) and half the time from different categories (*incongruent*). If selectivity fails and the observer therefore bases their judgment on the stimulus in the uncued location instead of on the stimulus in the cued location, it will result in an error in the incongruent condition but not in the congruent condition. If selectively fails completely, then performance in the incongruent condition should be at chance (50% in this two-choice task), whereas if selectivity is perfect, performance should be equal in the congruent and incongruent conditions. Thus, congruency effects (i.e., differences in performance in congruent and incongruent conditions) in a spatial filtering paradigm provide a measure spatial selectivity.

The Yigit-Elliot (2012) filtering experiment was conducted with disks of 0.7° diameter at an 8° eccentricity and used two separations between the relevant and irrelevant disks. The separations were 1° and 11° of visual angle, which is equivalent to polar angles around fixation of 6° and 90°. In other words, the disks were almost touching for the smallest separation and were half-way around the display from one another for the largest separation. For the small separation, accuracy was 98% for the congruent condition and 80% for the incongruent condition. The difference is a congruency effect of 18 ± 2%. In contrast, for the large separation, accuracy was 99.0% for the congruent condition and 98.5% for the incongruent condition, a congruency effect of 0.5 ± 0.1%. This result illustrates how congruency effects in spatial filtering are sensitive to separation. With small enough separations in this task, congruency effects should approach 50%. And the results of this experiment show that they fall to less than 1% with a large separation. To further quantify the selection process, we estimated the critical separation at which congruency effects were halfway between perfect and chance. In Palmer and Moore (2009), the critical separation was as a small as 1° for stimuli that were 8° in the periphery. Similar results were found in Yigit-Elliot et al. (2011).

The congruency effects observed in these spatial filtering experiments can be accounted for by errors of selection. However, there are at least two alternative types of selection error that are defined by their processing loci that could account for the effects, selection error within early perceptual processing or selection error within later processes after immediate perception. Palmer and Moore (2009) described a specific late selection-error hypothesis, referred to as *selection by decision*, that could account for the large effect of spatial separation on congruency effects that were found in the spatial filtering experiments. Imagine that two percepts are formed for two disks in two different locations. Each percept has a perceived location. These perceived locations are compared to the representation of the cued location. The percept with the location that is closest is selected for further processing to determine the response. Limited localization can cause a stimulus in an uncued location to be selected incorrectly for the required judgment. Such selection errors are most likely to occur when irrelevant stimuli appear in locations that are close to the cued location and are increasingly less likely to occur as the separation between the relevant and irrelevant stimuli increases. Congruency effects can range from 50% (chance) to 0% under this hypothesis.

A specific example of an early selection-error hypothesis is *imprecise targeting* described by Bahcall and Kowler (1999). Under the imprecise targeting hypothesis, in contrast to selection by decision, selection occurs *before* stimuli are presented. Specifically, a cued location is selected so that stimuli that appear in that location are processed fully and stimuli that appear in unselected locations are not. Spatial imprecision in the selection process can cause an uncued location to be selected incorrectly resulting in any irrelevant stimulus that appears in that location to be processed for the required judgment. Such selection errors are most likely to result in an irrelevant stimulus being processed when it appears in a location that is close to the cued location. The likelihood of an irrelevant stimulus being processed instead of a relevant stimulus decreases as the spatial separation between relevant and irrelevant stimuli increases. Again, congruency effects can range from 50% (chance) to 0% under this hypothesis.

To discriminate between different possible loci of error in spatial filtering we adapted the simultaneous-sequential paradigm (Shiffrin & Gardner, 1972) to the spatial filtering paradigm. Specifically, we compared performance with simultaneously presented relevant and irrelevant stimuli to performance with sequentially presented relevant and irrelevant stimuli (Palmer & Moore, 2017). If errors in spatial filtering arise from having to process both relevant and irrelevant stimuli simultaneously within immediate processing such as is maintained by the imprecise-targeting hypothesis, then there should be an advantage for the sequential condition over the simultaneous condition. Alternatively, if errors arise within some later process, such as is maintained by the selection-by-decision hypothesis, then there should be no advantage for sequential presentation, and therefore performance is predicted to be the same in the simultaneous and sequential conditions. To clarify this latter prediction, consider that by hypothesis, the cued and uncued disks are perceived equally well, and there is therefore no advantage provided by sequential presentation. The error comes later in processing when deciding about the two percepts (e.g., which one is closer to the cued location), which is unaffected by sequential versus simultaneous presentation. Results from this experiment confirmed that performance was similar in the simultaneous and sequential conditions indicating that the locus of errors in this spatial filtering task derive from later process such as selection by decision.

To further pursue the nature of spatial selectivity, we investigated whether errors on incongruent trials in spatial filtering arise from a graded process such as attenuation of representations of stimuli in uncued locations at some level of processing (Treisman, 1960) or an all-or-none process such as blocking representations of uncued items at some level of processing from accessing further processing (Broadbent, 1958). This was tested by varying the contrast of the relevant and irrelevant stimuli. A graded process like attenuation predicts that increasing the strength (i.e., the contrast) of an irrelevant stimulus can overcome its attenuation and therefore errors increase with increasing contrast of irrelevant stimuli. An all-or-none process like blocking, however, cannot be overcome by increasing contrast of the irrelevant stimuli. Therefore, an all-or-none model predicts that errors should asymptote with increasing contrast of the irrelevant stimulus. In three studies (Palmer & Moore, 2009; Yigit-Elliot et al., 2011; Yigit-Elliot, 2012), there was clear evidence that errors in a spatial filtering task were due to an all-or-none process such as blocking and not to a graded process like attenuation.

The set of studies reviewed in this section sketches a story of how spatial filtering works. Errors in spatial selection occurred with a critical separation of about 1° (at 8° eccentricity), whereas spatial selection was almost perfect at large separations. The errors occurred within a process that is later than immediate perception, such as in decision. And, finally, the errors arose due to an all-or-none mechanism such as blocking, rather than a graded process such as attenuation. The experiments ruled out the possibility that the errors occurred within any process that depended on relevant and irrelevant stimuli being present simultaneously, such would be expected if they were due to crowding or a perceptual capacity limit.

### A failure of spatial selectivity

While spatial selectivity is good for the widely separated stimuli in the cases just reviewed, there are cases in which spatial selectivity is not as good. Specifically, there are studies showing that tasks requiring divided attention do not show good spatial selectivity even for widely separated stimuli. Consider a dual task that requires separate judgments of two widely separated stimuli. For each individual judgment, one of the stimuli is relevant and the other is irrelevant. Thus, the individual judgments require spatial filtering. But because there are two judgments, both stimuli are relevant to the task as a whole. Consider as examples two studies that investigated such dual tasks.

The first example is Experiment 1 of Bonnel et al. (1992). They compared performance for detecting brief (20 ms) luminance increments in single and dual tasks and measured both dual-task deficits and congruency effects. An observer viewed two continuously illuminated LEDs to either side (left and right) of fixation. On a trial, each of these LEDs independently incremented in luminance or remained constant. For each LED, observers indicated if an increment occurred by a yes–no response and confidence judgment. There were many conditions, but we focus on comparing the single-task condition when only one stimulus was relevant and the dual-task condition with instructions to “equally allocate attention.” There was little or no difference in overall performance between the single and the dual tasks (no dual-task deficit). But what about congruency effects? For the single task, there was little or no congruency effect (77% vs. 78% correct for congruent versus incongruent responses, see their Table 2). For the dual task, however, there was a 15% congruency effect (82% vs. 67% correct for congruent vs. incongruent responses). Thus, despite widely separated stimuli, there were congruency effects for the dual task that were larger than those found for the single task. In a further experiment, they showed that this difference in congruency effects for single and dual tasks was also obtained for discriminating between increments and decrements, which do have dual-task deficits. These two sets of results are surprising: the lack of dual-task deficit suggests that there is no processing capacity limit for detecting two light increments at once. Moreover, the lights were so far apart that their locations should not be confusable. So why were there such large congruency effects? That is the question we seek to understand in the current study.

The second example is Experiment 1 of Logan and Gordon (2001). An observer viewed displays of two digits that were about 0.5° in height and were presented about 0.5° above and below the center of the display. The task was a magnitude judgment of each digit: press one key if the digit was less than “5” and another key if the digit was greater than “5” (the digit “5” was never shown). The digits were either presented simultaneously or sequentially but we focus on the simultaneous condition here. Observers were instructed to either make a single response to one of the digits (single-task condition), or to make two separate responses, one to each digit in turn (dual-task condition). In this experiment, the digits were displayed for one second and the primary measure was response time. (Accuracy was high and nearly constant at 95% correct for both single and dual-task conditions.) There were several results. First, there was a dual-task deficit. The overall mean response time was faster for the single task than the first response of the dual task (~ 575 ms vs. ~ 725 ms, respectively). What about congruency effects? For the single task, the congruency effect was near zero (~ 568 and 565 ms for congruent and incongruent conditions, respectively). For the dual task, the congruency effect was 60 ms for the first response (~ 695 ms vs. ~ 755 ms for congruent and incongruent, respectively), and the congruency effect was ~ 146 ms for the second response (~ 890 ms vs. ~ 1,036 ms for congruent and incongruent, respectively). Thus, there was a substantial congruency effect for the dual task and little or no congruency effect for the single task. In further experiments, Logan and Gordon showed a similar pattern of congruency effects for judgments of color patches and color words, and for judgments of pictures and words.

We selected these two examples because they required spatial filtering for the component tasks. There are similar examples from dual-task versions of the flanker paradigm which involves more than spatial filtering (Hubner & Lehle, 2007). A review of this larger context is deferred to the *General discussion*. To summarize, spatial filtering experiments that involve a single task show a high degree of spatial selectivity with little or no congruency effects for widely separated stimuli. In contrast, spatial filtering experiments that involve a dual task, show congruency effects even for widely separated stimuli.

We have been discussing the congruency effects in these studies as reflecting errors of selection. There is, however, an important alternative hypothesis to consider. Assuming parallel processing of the stimuli, there could be interactions, such as crosstalk between information channels, that cause congruency effects separate from any failures of selection. Such *interactive processing hypotheses* have been proposed as explanations for congruency effects in both the flanker paradigm (Eriksen & Eriksen, 1974) and in dual tasks (Hommel, 1998; Navon & Miller, 1987). Interactive processing explanations have also been described for other related domains including crowding in perception (e.g., Parkes et al., 2001), memory interference (e.g., Oberauer & Lin, 2017) and response priming (e.g., Morton, 1969). Interactive processing accounts are tested in Experiment 1 and discussed in the *General discussion*.

### Goals

To maximize the effects of divided attention on the spatial selectivity of spatial filtering, we used conditions in which filtering is nearly perfect for a single-task condition. Specifically, separate detection tasks were used for two widely separated stimuli. To foreshadow the results, when only one stimulus was relevant (single-task condition), there were little or no congruency effects. But when both stimuli were relevant (dual-task condition), there were substantial congruency effects which indicates a failure of spatial selectivity.

We asked three questions about the increased congruency effects found for dual-task conditions that are analogous to those that we asked about errors in our earlier spatial filtering studies. First, are these dual-task congruency effects due to selection error (Yantis & Johnston, 1990), interactive processing (Navon & Miller, 1987), or both? Second, is the locus of these dual-task congruency effects in immediate processes (e.g., stimulus-driven perceptual processes), in later processes (e.g., decision), or both? Third, are these dual-task congruency effects due to errors in a graded process (e.g., attenuation, Treisman, 1960) or an all-or-none process (e.g., blocking, Broadbent, 1958)? Together these three questions identify 18 kinds of theory (3 × 3 × 2). Thus, answering the individual questions with regard to specific stimulus and task conditions can begin to distinguish among alternative theories of spatial filtering tasks.

## General methods

### Overview

We investigated the detection of a simple visual pattern, a horizontal Gabor patch in visual noise. On every trial there were two stimuli presented, one on the left and one on the right of the point of central gaze fixation. Each stimulus was a square patch of dynamic noise which on a random half of trials contained a horizontal Gabor patch. The two stimuli were independent with regard to presence or absence of a target Gabor patch. Thus, they could be *congruent*, meaning that they both contained a target or both contained only noise, or *incongruent*, meaning that one contained a target and the other did not. At the end of each trial participants were post-cued to judge one stimulus at a time: was there was a Gabor patch in it or just noise. Participants reported their judgment along with a confidence rating. Our primary comparison was between a dual task condition and a single task condition. The stimuli were the same in these two conditions. The difference was that in the single-task condition, only one stimulus was task-relevant. One side was pre-cued, and the participant only had to make a response about the stimulus on that side, so they could focus spatial attention on that side. In the dual-task condition, both stimuli were task-relevant because either both had to be responded to (Experiment 1), or which of the two stimuli had to be responded to was cued only after the stimuli were gone (Experiment 2). Thus, the dual-task condition requires divided attention. It is important to note that in the “dual-task” condition, participants made *the same judgment* (presence or absence of a Gabor patch) about two independent stimuli. Our key question regards how much the congruency of the two stimuli affected task accuracy in the single-task and dual-task conditions.

### Stimuli

The stimuli were either dynamic noise alone or dynamic noise with a briefly presented single horizontal Gabor patch. Observers judged the presence or absence of the Gabor patch. The Gabor patch was always horizontal with the grating component in sine phase (i.e., the grating was at zero at the center of the patch) and a spatial frequency of 1 c/d. The envelope component was a Gaussian with a standard deviation of 0.5°. It was truncated to a maximum size that was four times the Gaussian standard deviation (4 × 0.5° = 2°). The contrast of the Gabor patch was adjusted by the experimenter during practice to achieve overall performance around 75–85% correct for each observer. The resulting contrast values ranged from 18 to 35%.

The Gabor patch was presented briefly with temporal uncertainty during the relatively long dynamic noise display. Specifically, the Gabor contrast was modulated by a Gaussian temporal waveform that had its peak during the noise display and a standard deviation of 0.05 s. The peak was restricted to not occur in the first or last 0.2 s of the display. Consequently, the effective duration of this Gabor was about 0.1 s. This is much shorter than the noise display duration of 1.0 s. The onset of the target was the same for the two tasks to prevent the strategy of switching the attended side after seeing one target. This synchrony of target presentation was the only way in which the physical stimuli for the two tasks were dependent on one another.

### Procedure

**Fig. 1:**
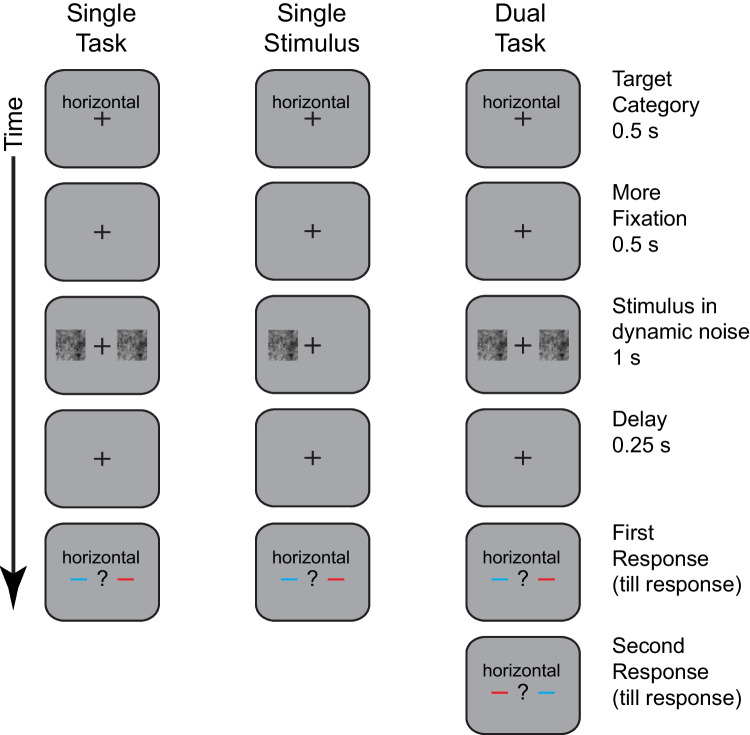
An illustration of the general procedure. The stimulus sequence is shown for the three main conditions: single task, single stimulus, and dual task. All conditions begin with a fixation display (along with a word reminding the subject to look for horizontally-oriented targets). After a brief delay, the stimuli are displayed within a 1-s movie of dynamic 1/f noise. Then after a delay, the observer is prompted for a response using a response prompt that specifies the relevant side of display for this response (the red line for some subjects, blue for others). In the single-task condition, the relevant side is blocked and the observer is informed at the beginning of the block. In the single-stimulus condition, everything is the same except that there is no stimulus or noise on the irrelevant side. In the dual-task condition, both sides are relevant for every trial of a block. The display sequence is identical to the single-task condition, but with both sides tested in sequence

The procedure is illustrated in Fig. 1, which shows the stimulus sequence for the three conditions of the first experiment. Consider first the *single-task condition* in the left column. A trial began with a fixation cross and a word by indicating the relevant target’s orientation (“horizontal”). The target was always horizontal, but the label was included because this experiment was run alongside other experiments with semantic categorization of words that will be published separately. Observers were instructed to maintain fixation and it was enforced by monitoring eye position on all trials. After a brief interval, a display of dynamic noise was presented for 1 s. During that time, a Gabor patch might be presented with a duration of about 0.1 s (see *Stimulus* section). There were noise displays on both sides, and the relevant stimulus was always on one side for a given block of trials. Thus, the observer's task was to judge just one side (hence *single-task condition*). After a short interval to avoid masking, there a post-cue prompted the observer to respond. The post cue consisted of two colored-lines, one to the left of fixation and one to the right. Each observer was assigned a cue color (red or blue) and was to respond according to the stimulus on the side that had the line with the cued color. For the example illustrated in the figure, the relevant cue is blue. It is on the left and accordingly an observer is to respond to the stimulus on the left. This arbitrarily assigned color cue was used so that there would be no stimulus differences between the left and right sides. As noted, for the single-task condition, the post-cue indicated the same side on every trial of a given block. The trial ended with a key-press response in the form of a confidence rating and tone feedback was given for errors. Of particular interest was the effect of congruency between the relevant stimulus and the irrelevant stimulus.

Next consider the *dual-task condition* shown in the rightmost column. The displays were identical to those of the single-task condition up to the response prompt. In the dual-task condition responses were required for both stimuli. The post-cue indicated which of two stimuli to respond to. In Experiment 1, both stimuli were cued, one after the other. In Experiments 3 and 4, only one stimulus was cued for a response but which would be cued was unpredictable and therefore both stimuli were relevant. Of particular interest was the effect of congruency between the two relevant stimuli.

The third *single-stimulus condition* is shown in the middle column of the figure. The task was the same as with the single-task condition: Judge an entire block of trials with the relevant displays on one predictable side. The distinctive feature was to remove the irrelevant display. This allows one to test if the presence of an irrelevant display has any effect on performance. This would reveal a failure of selective attention that can reduce performance.

**Fig. 2:**
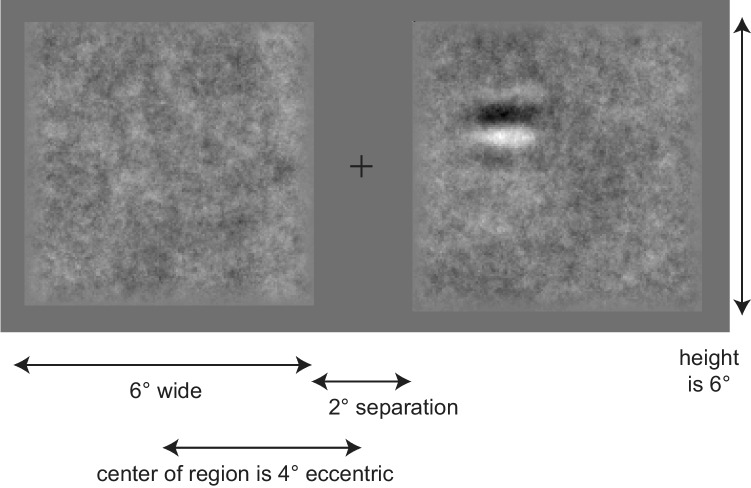
An illustration of a single frame of the stimulus display. Two examples of the 1/f noise are shown on each side of fixation. The display on the right includes a high contrast Gabor patch (80%). The figure also specifies the dimensions of each display element

The spatial structure of the display is shown in Fig. 2. The two noise movies were 6 × 6° to either side of a 0.5° fixation cross. They were each centered at an eccentricity of 4° which resulted in a 2° space between them. Overall, the two noise movies filled the middle 14° of a video monitor that had a viewable width of about 32°. An example Gabor patch is shown in the right side with a contrast of 80%, which is much higher than used in all but the last experiment. It was presented with spatial and temporal uncertainty in the noise display. For example, the Gabor patch had a Gaussian envelope with a standard deviation of 0.5°. This made them effectively about 1° in size. The Gabors were excluded from near the edge of the display (< 0.5°) to prevent clipping the Gabor, and the noise was attenuated to prevent sharp edges. As a result, the center of the Gabors appeared anywhere in a region of 5 × 5° (25 square degrees).

The dynamic noise had spatial and temporal frequencies with amplitudes inversely proportional to frequency (1/f noise). Individual pixels had luminance values that were initially independently sampled from a Gaussian distribution and were then filtered in space and time so that each dimension had an amplitude at each frequency that was inversely proportional to the frequency. The luminance values of each pixel had a distribution with a mean at zero contrast and a standard deviation of 12% contrast. New noise frames were presented at a rate of 30 Hz (every 4 th refresh of the 120-Hz display). In summary, the contrast for component frequencies varies inversely with the frequency. Thus, the noise has relatively more low frequency content than white noise. This kind of noise is useful because it equates the “power” per octave which is more relevant to human vision than equating the power per degree as in white noise (Field, 1987). Thus, 1/f noise is believed to be effective noise for reducing the visibility of stimuli with a wide range of spatial and temporal scales.

Congruency was always defined with regard to the left and right displays, whether they were both relevant (dual task) or only one was relevant (single task). Specifically, congruent trials were either trials in which there was a horizontal Gabor on both sides or there were noise-alone stimuli on both sides. Incongruent trials were ones in which there was a horizontal Gabor on one side and a noise-only stimulus on the other. All Gabor targets were identical in orientation and contrast, but they varied in position, in their time of onset, and in the surrounding noise pattern.

The three main conditions (dual-task, single-task, single-stimulus) were blocked. In addition, the side for the single-task and single-stimulus conditions was blocked. This yielded five kinds of blocks: dual-task; left-single-task; right-single-task; left-single-stimulus; right-single-stimulus. To equate the number of trials in the primary conditions, there were 2 dual-task blocks along with one each of the four other kinds of blocks.

### Analysis

Observers responded with one of four key presses that indicated likely-no, guess-no, guess-yes, or likely-yes. These ratings were used to form a receiver operating characteristic (ROC) function and performance was summarized by the percent area under the ROC (*A*_*ROC*_). For reasonable assumptions, this *A*_*ROC*_ measure is equivalent to the percent correct measured by a forced choice paradigm (Green & Swets, 1966). To estimate *A*_*ROC*_ the simple trapezoid method was used to avoid making distributional assumptions (Macmillan & Creelman, 2005).

Each result was described with several statistics: the standard error of the mean based on that sample alone, the results of the relevant hypothesis test, and 95% confidence intervals. Each hypothesis test was done as a planned contrast based upon a condition-by-subject, within-subject ANOVA. Our primary analysis was the congruency effects and the difference between congruency effects for dual and single tasks. We used one-tailed tests to gain sensitivity given that negative results were unexpected. For all secondary analysis, we used two-tailed tests.

### Aspects of the procedure motivated by our imaging experiments

Two aspects of this procedure were intended to increase the size of a functional magnetic resonance imaging (fMRI) signal examined in a separate study (White et al., 2017). The spatial extent of the noise display was relatively large (6 × 6°) and nearly all of it is relevant to the judgment due to the spatial uncertainty of the target. The duration of the noise display was relatively long (1 s) and nearly all of it is relevant due to the temporal uncertainty of the target. In summary, the large and long noise displays provided a potent signal for our related fMRI study.

### Observers

In each experiment there were six observers. Many were in multiple experiments and over the series of experiments there were a total of 11 observers. Some were unpaid volunteers and others were paid $20/h. All had normal or corrected-to-normal vision. Each gave informed consent in accordance with the University of Washington Institutional Review Board in adherence with the Declaration of Helsinki.

To determine the appropriate sample size, we used data from two previous spatial filtering experiments that had measured congruency effects (Yigit-Elliot et al., 2011, Experiment 1; Yigit-Elliot, 2012, Experiment 2.2). These studies varied contrast widely so performance varied from chance to perfect. From this range, conditions were selected that had similar performance levels as the current study (70–90% correct). In addition, the number of selected trials was similar to the current study (~ 300 congruent and ~ 300 incongruent trials). For the selected conditions from the two experiments, the standard deviation of the congruency effect was 2.62% and 4.96% for an average of 3.79%. Based on this variability, detecting a congruency effect of 5% with 80% power in a one-tailed t-test required a minimum sample of *n* = 6. To further evaluate this choice, we did a post hoc analysis based on the current experiments. For Experiments 1, 2, and 3, the standard deviation of the congruency effects observed for both single- and dual-task conditions had a grand mean of 3.68%. Based on this standard deviation, detecting a congruency effect of 5% with 80% power in a one-tailed t-test also required a minimum sample size of *n* = 6. Thus, the sample size was adequate to detect a congruency effect of 5%.

### Display apparatus and eye-movement monitoring

The stimuli were displayed on a flat-screen CRT monitor (19-in. ViewSonic PF790) controlled by a Power Mac G4 (Dual 1.0 GHz) using Mac OS X 10.6.8. The experiment was displayed at a resolution of 832 × 624 pixels, a viewing distance of 60 cm (25.5 pixel/degree at screen center), and a refresh rate of 120 Hz. The monitor had a peak luminance of 119 cd/m^2^, and a black level of 4.1 cd/m^2^, mostly due to room illumination. Stimuli were displayed using Psychophysics Toolbox 3.0.11 for Matlab R2012a (Brainard, 1997). A chin rest with an adjustable chair ensured a fixed distance to the display.

On all trials, eye position was recorded using an EyeLink II, 2.11 with 250-Hz sampling (SR Research, ON). The EyeLink II is a head-mounted binocular video system and was controlled by software using the EyeLink Developers Kit for the Mac 1.11.1 and the EyeLink Toolbox 3.0.11 (Cornelissen et al., 2002). The position of the right eye was recorded for all trials, and trials were included in the analysis only if fixation was confirmed. When fixation failed, five consecutive high frequency tones were sounded and the trial was aborted. The percentage of aborted trials for each observer in each experiment ranged from 0.5% to 4.4% with an overall mean including all experiments of 2.0 ± 0.2%. Thus, the observers maintained fixation on almost all trials and none of the analyses included trials with blinks or saccades to the stimuli.

### The importance of randomized response order

We have employed a refinement intended to help isolate the role of perception in divided attention effects. Specifically, response prompts on dual-task trials indicate which response to make, in an unpredictable order (left then right or right then left). Using such a response prompt prevents an unintended prioritization of one response over the other. For example, it can prevent effects due to preparing the first response while still perceiving the other stimulus. In a previous study (Ernst et al., 2012), we found in pilot work that there was an order effect when the responses were in a fixed order but not when using an unpredictable order. Such fixed order cues might have contributed to finding dual-task deficits in some previous studies of simple detection tasks (e.g., Pastukhov et al., 2009). A related finding was reported recently showing that dual-task deficits in a speeded dual task were reduced when the tasks occurred in an unpredictable order compared to in the same order across trials (Lyphout-Spitz et al., 2024).

## Experiment 1

In this experiment, we measured the effect of divided attention on spatial selectivity. In addition, we began to distinguish between theories of selection error versus interactive processing as explanations for congruency effects.

### Methods

In the first experiment, congruency effects and dual-task deficits were measured for detecting Gabor patches. As just described, there were three blocked conditions: single task, single stimulus, and dual task. In addition, the data from the dual-task condition were broken down by the first or second response. There were six observers who, after practice, participated for five hour-long sessions resulting in 640 trials in each of the four main conditions for each observer.

### Results

#### Congruency effects

**Fig. 3:**
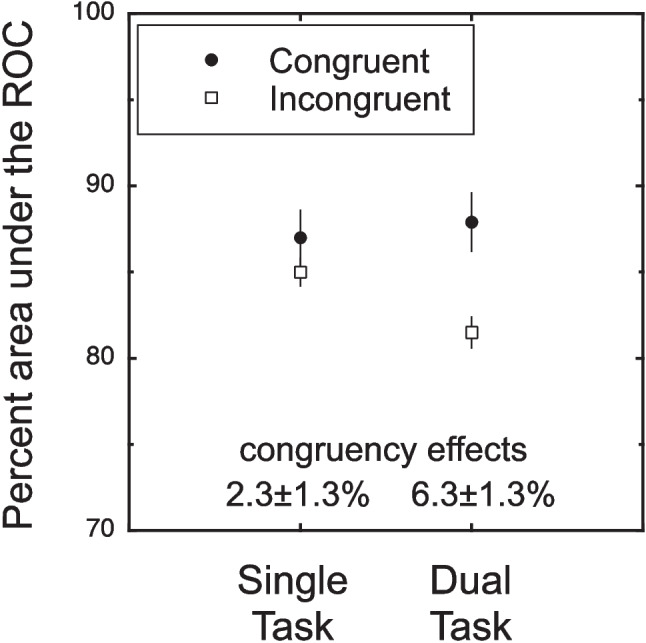
Results of Experiment 1. The percent area under the receiver operating characteristic curve (ROC) is plotted for the single-task and dual-task conditions. This measure can be thought of as an unbiased percent correct (Green & Swets, 1966). These conditions are further broken down by whether the trial had congruent (solid disk) or incongruent (open square) stimuli. The congruency effect (difference between congruent and incongruent) was larger for the dual-task condition compared to the single-task condition

For all of the results, performance was measured in terms of the percent area under the ROC function. As described in the methods, this measure can be thought of as an estimate of the unbiased percent correct. Our primary interest are the congruency effects. The stimuli were congruent if they were associated with the same response. The effect of congruency is shown in Fig. 3 for the single-task and the dual-task conditions. These conditions are broken down by whether the trial had congruent (solid disk) or incongruent (open square) stimuli. The statistical analyses are planned contrasts for the congruency effects using a common error term based on a condition-by-subject, within-subject ANOVA (*F*(3,15) = 9.30, *p* = 0.001). For the single task, the congruency effect was relatively small and not significant (2.3 ± 1.3%, 95% CI − 0.5, 5.2, *t*(15) = 1.74, *p* = 0.051, one tailed). In contrast, the dual-task congruency effect was larger and significant 6.3 ± 1.3% (95% CI 3.5, 9.2, *t*(15) = 4.72, *p* < 0.001 one tailed). The difference between the congruency effects in the dual and single tasks was also significant (4.0 ± 1.9, 95% CI − 0.04, 8.0, *t*(15) = 2.11, *p* = 0.026, one tailed). Thus, congruency effects were larger for the dual-task condition than the single-task condition. A further analysis of the ROC underlying the area measure is presented in Appendix A. It provides additional evidence that the congruency effect is due to changes in sensitivity and not bias.

#### Dual-task deficits

To give the most sensitive measure of dual-task deficits, we combined the single-task and single-stimulus conditions and the two responses in the dual-task condition. The difference between aggregated single-task and dual-task conditions was 1.4 ± 0.7%, which was not significant (95% CI − 0.4, 3.2, *t*(5) = 1.97, *p* = 0.106, two tailed). Thus, there was little dual-task deficit for Gabor detection. This lack of a dual-task deficit contrasts with large dual-task deficits found for other stimuli under similar conditions (e.g., up to 15% effects with masked words; White et al., 2018, 2020). This was expected based on results from prior dual-task studies using detection judgments which found little to no dual-task deficit (e.g., Bonnel et al., 1992; White et al., 2017).

#### Secondary effects

We also describe three secondary effects to provide context. First, the difference between the single-task and single-stimulus conditions was near zero and not significant (0.1 ± 1.0%, 95% CI − 2.4, 2.5, *t*(5) = 0.09, *p* > 0.1, two tailed). This is consistent with near perfect selection and no interference between stimuli in the single-task condition, as expected with a large separation between stimuli (e.g., Palmer & Moore, 2009). Second, the difference between the first and second responses for the dual-task condition was also near zero, and is not significant (− 0.7 ± 0.5%, 95% CI − 2.0, 0.5, *t*(5) = 1.47, *p* > 0.1, two tailed). This is consistent with no memory or response interference that was worse for the second response compared to the first. Third, we measured the correlation between the two responses on a single trial. Parallel and serial models make different predictions about such correlations (Sperling & Melchner, 1978). Typical serial models predict negative correlations between a correct response on one task and a correct response on the other task. Typical parallel models in themselves predict no correlation. But, any common noise source for the two tasks would introduce a positive correlation. In this experiment, there was a small but significant positive correlation of 0.046 ± 0.015, (95% CI 0.007, 0.085, *t*(5) = 3.01, *p* = 0.030, two tailed). One can also consider the correlation broken down by the kind of trial. For target-target trials it was 0.05 ± 0.03. For target-distractor trials, it was − 0.01 ± 0.01. And for distractor-distractor trials, it was 0.18 ± 0.07. Thus, trials with two distractors and no targets had the largest positive correlation. This pattern of correlations was also found for the color tasks in White et al. (2018). In summary, there was no sign of the negative correlation expected from a serial model. For dual-task experiments that find such negative correlations, see Sperling and Melchner (1978) or White et al., (2018, 2020).

### Discussion

The primary result of this experiment was that the congruency effect was larger in the dual-task condition than in the single-task condition. This confirms previous results showing that congruency effects in spatial filtering tasks are magnified under conditions of divided attention (Bonnel et al., 1992; Logan & Gordon, 2001).

Consider possible interpretations of this finding. In this experiment, the single-task and dual-task blocks differed only in the knowledge of which stimulus would be post-cued. Because the stimuli were identical, any stimulus-driven process must also be identical. Thus, any interactive processing that is a function of the stimuli, and not top-down control, does not predict the congruency effect being specific to the dual-task condition. In contrast, the results are consistent with the either errors in selection or an account that combines selection and interactive processing. We consider further these two possibilities in the *General discussion*. In summary, the use of identical displays in both the single- and dual-task conditions allows us to reject a pure, stimulus-driven interactive processing account of the congruency effects.

## Experiment 2

In this experiment, we tested whether the congruency effects in the Gabor detection task are due to immediate stimulus-driven processes, such as perception or memory encoding, or to later processes, such as memory maintenance, retrieval, or decision, which are not dependent on the continued presence of the stimuli. The approach was to compare a condition in which the stimuli are presented simultaneously, as they were in Experiment 1, to a condition in which they are presented sequentially. Any effects that are dependent on immediate stimulus-driven processes should be reduced or eliminated in the sequential condition. This strategy has been used extensively in the visual search literature, which is an alternative approach to studying divided attention (Scharff et al., 2011a, 2011b; Shiffrin & Gardner, 1972), but less often with dual tasks (cf. Duncan, 1980; Logan & Gordon, 2001).

### Methods

**Fig. 4:**
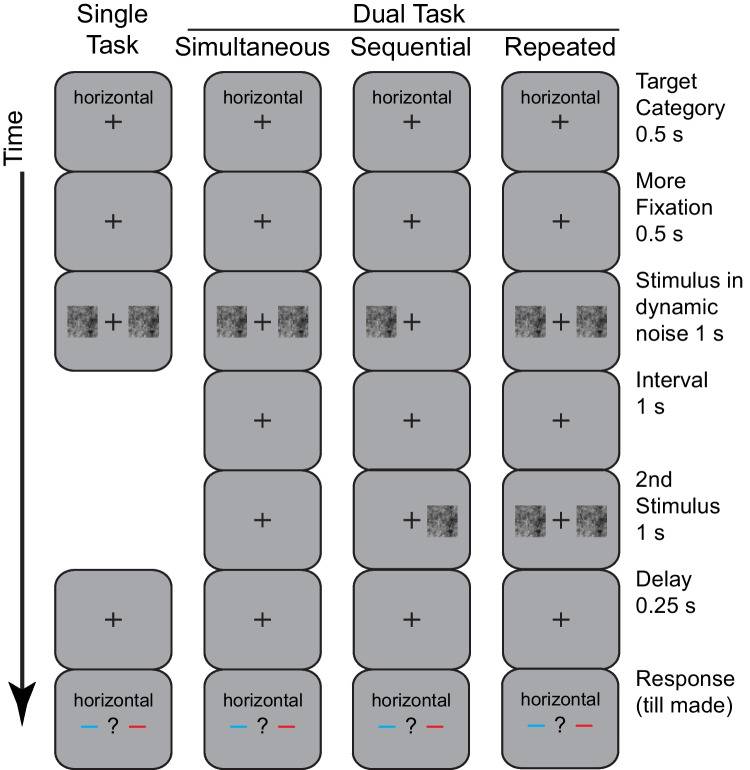
Illustration of the procedure of Experiment 2. The stimulus sequence is shown for the four conditions: single task, simultaneous dual task, sequential dual task, and repeated dual task. All conditions have the same initial and ending displays as the previous experiments. The single-task and simultaneous dual-task conditions are the same as the previous experiments. In the sequential-dual-task condition, the stimuli for the left and right sides are presented in separate displays with an intervening interval of 1 s. In the repeated-dual-task condition, the display for the simultaneous dual-task condition is repeated in a second display

Experiment 2 combined the dual-task and single-task conditions from Experiment 1 with two new conditions as shown in Fig. 4. The four conditions are in separate columns. The leftmost column is the single-task condition which is unchanged from Experiment 1. Recall that the relevant stimulus (left or right) was the same for all trials within a given block. The second column is the simultaneous dual-task condition. This was also unchanged except that the participant only had to make one response to judge the stimulus on one side. They still were required to attend to both stimuli, because they did not know in advance which side would be post-cued.

The third condition is the *sequential dual task*. The new feature is that the critical stimulus display is split into a pair of displays shown in sequence. In this example, the left-side stimulus was shown first and the right-side stimulus was shown second. This order was constant within a block but varied across blocks. The duration of the individual displays was unchanged (1 s). The interval from the end of the first display to the beginning of the second display was 1 s, which is more than sufficient to shift attention from one side to the other (e.g., Moore et al., 1996; Ward et al., 1996). If the dependency between the tasks is specific to immediate stimulus-driven processing, then the congruency effect should disappear in sequential condition because the stimuli are never present at the same time. One can think of the sequential condition as being equivalent to a sequence of single-task conditions. On the other hand, if the dependency is not due to immediate stimulus-driving processes, but is instead due to some later aspect of processing, then the congruency effect should not differ between the simultaneous and sequential conditions.

The fourth condition is the *repeated dual task*. It also had two sequential displays. But these displays repeat the entire simultaneous display rather than split them apart. This purpose of adding this condition is to provide a comparison for the expected size of the dual-task deficit. For a class of fixed-capacity models, the difference in performance between dual and single tasks (dual-task deficit) is predicted to match the difference between the repeated and simultaneous dual-task condition (Scharff et al., 2011a, 2013). To get an intuition of this, assume a serial model that can process only a single stimulus in the brief displays of this experiment. A dual-task deficit arises because in the dual-task condition, only one of the two stimuli can be processed whereas in the single-task condition, there is only one relevant stimulus so being able to process one stimulus is sufficient. Similarly, the repeated effect arises because only one of the two stimuli can be processed in the simultaneous dual-task condition while both of the stimuli are processed in the repeated dual-task condition. In other words, the repeated condition gives an observer a second chance at the second stimuli which, in the extreme, makes it as good as the single-task condition.

In summary, this experiment combined the dual-task paradigm with a comparison of simultaneous and sequential displays. There were four main conditions: single task, simultaneous dual task, sequential dual task and repeated dual task. There were six observers who, after practice, participated for 7 h resulting in 672 trials in each of the four main conditions for each observer.

### Results

#### Congruency effects

**Fig. 5:**
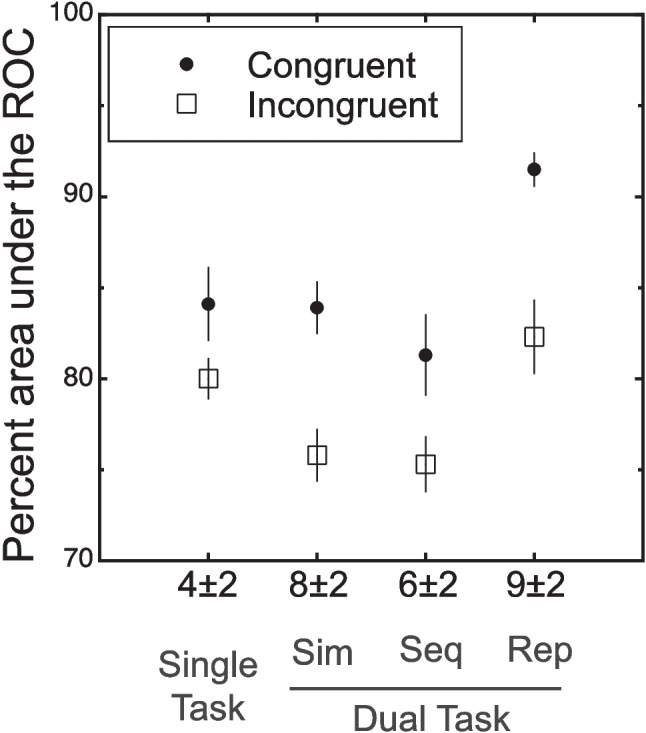
Results of Experiment 2. The percent area under the receiver operating characteristic curve (ROC) is plotted for four conditions: single task, simultaneous dual task (sim), sequential dual task (seq), and repeated dual task (rep). These conditions are further broken down by whether the trial had congruent (sold disks) or incongruent (open squares) stimuli. There were significant congruency effects for all conditions

In Fig. 5, the four conditions are broken down by congruency and the values of each congruency effect are given at the bottom of the figure. As before, we used planned contrasts based on the error term for a condition-by-subject, within-subject ANOVA (*F*(7,35) = 21.00, *p* < 0.001). There were significant congruency effects in all conditions: the single-task congruency effect was 3.8 ± 1.6%, (95% CI 0.6, 7.1, *t*(35) = 2.39, *p* = 0.011, one tailed); the simultaneous congruency effect was 8.5 ± 1.6%, (95% CI 5.3, 11.7, *t*(35) = 5.32, *p* < 0.001, one tailed); the sequential congruency effect was 6.1 ± 1.6%, (95% CI 2.8, 9.3, *t*(35) = 3.79, *p* < 0.001, one tailed); and the repeated congruency effect was 9.0 ± 1.6%, (95% CI 5.8, 12.2, *t*(5) = 5.64, *p* < 0.001, one tailed). The single-task effect was smaller than the other effects. For example, it was half of the effect for the simultaneous dual task (3.8 vs. 8.5) and this difference was significant (4.7 ± 2.3, 95% CI 0.1, 9.3%, *t*(35) = 2.07, *p* = 0.023, one tailed). Recall the congruency effects in Experiment 1 with similar stimuli were 8% for the dual-task condition and 2% for the single-task condition. Combining over both experiments there were four dual-task conditions that on average had a congruency effect of about 8% and two single-task conditions that on average had a congruency effect of about 3%. Thus, for both experiments there was a larger congruency effect for dual tasks relative to single tasks.

The focus of this experiment is the sequential dual-task condition. For three of the four conditions, the stimuli were presented simultaneously. The sequential condition was different. Now the stimuli were presented sequentially with a full second between displays. If the congruency effect depends on immediate stimulus-driven processing, then it should be absent in the sequential condition. In fact, there was a significant 6.1 ± 1.6% congruency effect in the sequential condition. Thus, the results are consistent with the congruency effect being mediated by some later process such as memory maintenance, retrieval, or decision rather than immediate processing such as perceptual processes. Another possibility is that both immediate stimulus-driven and later processes contribute to the congruency effect. In that case, the congruency effect in the sequential condition should be smaller than the congruency effect in the simultaneous condition. Although this was true numerically, the difference was not significant (2.5 ± 2.3%, 95% CI − 2.1, 7.0, *t*(35) = 1.08, *p* = 0.29, two tailed).

#### Secondary effects

Overall performance in the four conditions was 82.1 ± 1.4%, 79.9 ± 1.2%, 78.3 ± 1.3%, and 87.1 ± 1.3% for the single, simultaneous, sequential, and repeated conditions, respectively. In words, performance was similar for the single, simultaneous, and sequential conditions and better in the repeated condition. Consider the two most relevant paired comparisons: the dual-task deficit (single-vs.-simultaneous) was 2.3 ± 0.5% which was significant (95% CI 1.1, 3.5, *t*(5) = 5.00, *p* = 0.004, two tailed); and, the sequential effect (sequential-vs.-simultaneous) was − 1.6 ± 0.8%, which was not significant (95% CI − 3.6, 0.4, *t*(5) = 2.02, *p* = 0.099, two tailed). Thus, there were small dual-task deficits and sequential effects that were in opposite directions. By comparison, in Experiment 1 the dual-task deficit was 1.4 ± 0.7% and not significant. An additional experiment described shortly also shows the dual-task deficit to be about 2%. Thus, the experiments in this article are consistent with a dual-task deficit of about 2% for Gabor detection. This is small relative to the 7% repeated effect, and the 8% dual-task deficit predicted by the fixed-capacity, parallel model for this performance level (Scharff et al., 2011a). We suggest that the dual-task deficit is probably not completely absent for Gabor detection, but it is small relative to these other standards. In contrast, performance was reliably better for repeated dual task. The repeated effect (repeated-vs.-simultaneous) was 7.2 ± 0.5%, (95% CI 6.0, 8.4, *t*(5) = 15.47, *p* < 0.001, two tailed). This effect confirms that an additional exposure to the display can improve performance. Thus, there is no ceiling on performance that is limiting the dual-task deficit.

### Discussion

The primary result of Experiment 2 was that dual-task congruency effects occur for sequential conditions as well as simultaneous conditions. It replicates similar results found in Logan and Gordon (2001) for quite different tasks. This result is consistent with the locus of the congruency effect being due to later processes rather than immediate stimulus-driven processes. If the only locus had been in immediate stimulus-driven processes, then the congruency effect should have been eliminated in the sequential condition. If the locus were in both immediate stimulus-driven processes and later processes, then most models predict larger effects in the simultaneous condition relative to the sequential condition. The small numerical difference in that direction was not significant.

## Experiment 3

This experiment was similar to Experiment 1 but with three changes to the procedure that were made in order to minimize sources of confusion that might cause the congruency effects. Specifically, we made it easier for the subjects to respond to the two stimuli independently in the dual-task condition – most importantly, by having two separate sets of response keys. The question is whether the congruency effects persist.

### Methods

There were two conditions: a single-task condition and a dual-task condition with just one response. The details were the same as Experiment 1 with the following modifications:

1. Separate keys were used for the two sides. Using a separate small keypad, the four keys on the left edge were assigned to the left-side task and the four keys of the right edge were assigned to the right-side task. For both tasks, the relevant four keys were arranged vertically and from bottom to top referred to the same confidence levels as in Experiment 1: likely-no, guess-no, guess-yes, likely-yes. This arrangement minimized Simon effects and eliminated decision errors in which one attempted to respond to one side when prompted to the other.
2. Observers were instructed to emphasize accuracy and take their time. To encourage that, the prompt following the stimulus display was delayed for 1 s instead of the 0.25 s in previous experiments.
3. The nature of independence between the two responses was discussed with each observer. Specifically, the two-by-two contingency table of possible stimuli for each task was explained and it was emphasized that they should make the two decisions independent of one another.

There were six observers who, after practice, participated for 7 h resulting in 1,344 trials in each of the two main conditions for each observer.

### Results and discussion

**Fig. 6:**
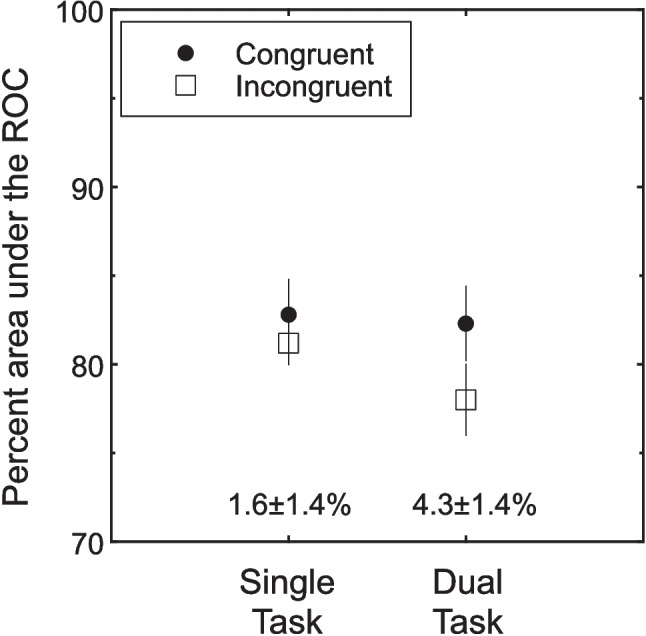
The congruency effects of Experiment 3. Percent area under the receiver operating characteristic curve (ROC) is plotted for single-task and dual-task conditions. The congruency effects were larger for the dual tasks than the single tasks

The effect of congruency is shown in Fig. 6 for the two main conditions with the values of each congruency effect given at the bottom of the figure. The following planned contrasts were based on the error term from a condition-by-subject, within-subject ANOVA (*F*(3,15) = 4.61, *p* = 0.018). For the single-task condition, the congruency effect was not significant (1.6 ± 1.4%, 95% CI − 1.5, 4.6, *t*(15) = 1.11, *p* = 0.143, one tailed). The congruency effect in the dual-task condition, however was significant (4.3 ± 1.4%, 95% CI 1.3, 7.4, *t*(15) = 3.03, *p* < 0.004, one tailed), though it was not significantly larger than for the single task (2.8 ± 2.0%, 95% CI − 1.6, 7.1, *t*(15) = 1.36, *p* = 0.097, one tailed). Overall, the pattern of congruency effects was similar to the prior experiments: a robust congruency effect for the dual-task condition and little to no effect for the single-task condition. Finally, the dual-task deficit was 1.8 ± 0.8% which was also not significant (95% CI − 0.3, 4.0, *t*(5) = 2.19, *p* = 0.080, two tailed). This is similar to what was found in the prior two experiments. In summary, the congruency effect is still present even when subjects can respond to the two stimuli with separate hands, and are fully informed and encouraged to judge the two stimuli independently.

## Pooled analyses of experiments 1–3

Because there was some variability across experiments regarding which congruency effects were statistically significant and different from each other, we conducted a pooled analysis that combined data across the three relevant experiments. Across Experiments 1, 2, and 3, there were 11 unique observers. For observers in multiple experiments, we averaged their data. As before, we did planned comparisons based on a condition-by-subject within-subject ANOVA, *F* (3,30) = 16.43, *p* < 0.001). For the single task, the congruency effect was relatively small but significant (2.5 ± 1.1%, 95% CI 0.4, 4.7, *t*(30) = 2.39, *p* = 0.023, two tailed). For the dual task, the congruency effect was larger and significant (6.6 ± 1.1%, 95% CI 4.4, 8.8, *t*(30) = 6.22, *p* < 0.001, two tailed). The difference between the single-task and dual-task congruency effects was also significant (4.1 ± 1.5%, 95% CI 1.0, 7.1, *t*(30) = 2.71, *p* = 0.001, two tailed).

This pooled analysis reinforces the results described in the separate experiments. Most importantly, dual-task congruency effects are significantly larger than single-task congruency effects, and single-task congruency effects while small, are significantly above zero.

## Experiment 4

Finally, we turn to the type of process underlying the congruency effect. Specifically, we distinguish between accounts that depend on all-or-none processes and accounts that depend on graded processes. This was done by comparing congruency effects with high-contrast stimuli to congruency effects to the congruency effects that occurred with the low-contrast stimuli of the previous experiments. This strategy is similar to that of Palmer and Moore (2009). When there is only one stimulus, detection is expected to approach perfect at high contrasts. With multiple stimuli, however, how performance is predicted to change with increasing contrast depends on one’s theory of congruency effects. Models in which congruency effects derive from a graded process such as attenuation, which can be overcome by increasing contrast, predict that congruency effects disappear at high contrasts. In contrast, models in which congruency effects derive from an all-or-none process, such as blocking, which cannot be overcome by increasing contrast, predict that congruency effects to persist even at high contrast. An example of a specific model with a graded process is the weighting model described in Appendix B. An example of a specific all-or-none model is the substitution model described in Appendix B.

### Methods

This experiment included single-task and dual-task conditions for Gabor detection. The new feature was to use stimuli with 80% contrast rather than the 18–35% contrast used in the prior experiments. Otherwise, the details of the experiment follow those of Experiment 3 (e.g., separate keys for the left and right tasks). There were six observers who participated for 4 h resulting in 192 trials in each of the main conditions. All had previous experience in at least one of the other experiments.

### Results

The percent area under the ROC was 99.7 ± 0.2% in the single-task condition and was 99.6 ± 0.4% in the dual-task condition. Congruency effects were very small and not significant in both conditions. For single tasks, they were − 0.5 ± 0.4% and for dual tasks they were 0.5 ± 0.4%. Such a near zero congruency effect is consistent with typical graded models and not consistent with typical all-or-none models (see Appendix B). As described in the appendix, all-or-none selection can prevent perfect performance even for highly visible stimuli. The dual-task deficit was 0.2 ± 0.2% and not significant. In fact, four of the six observers were perfect on all trials of both conditions.

### Discussion

In this experiment, we measured congruency effects for detecting a high contrast Gabor patch. In both single-task and dual-task conditions, performance was essentially perfect and there were no significant congruency effects. For a weighting model, which is a specific graded model that is described in Appendix B, congruency effects are predicted to decline as performance approaches perfection. In contrast, for a substitution model, which is a specific all-or-none model that is described in Appendix B, performance in the incongruent condition can never be perfect and the congruency effect grows with contrast. Thus, these results were consistent with a graded model and not with an all-or-none model.

Notice that finding evidence that is consistent with congruency effects being due to a graded process, rather than to an all-or-none process, contrasts with previous evidence from spatial filtering experiment with stimuli at small separation in which the evidence was consistent with selection errors occurring due to an all-or-none process and not with a graded process (Palmer & Moore, 2009; Yigit-Elliot et al., 2011). In those experiments, unlike Experiment 4, the effects remained, even for clearly visible high-contrast stimuli. This contrast is discussed more as a motivation for the working hypothesis that we offer in the *General discussion*.

## General discussion

### Summary of main results

In the four experiments presented above, we investigated how participants’ ability to respond selectively to just one stimulus depends on whether their attention is focused on one location or divided between two locations. We measured selectivity using congruency effects: impaired accuracy when relevant and irrelevant stimuli are associated with different responses instead of the same response. These congruency effects were consistently larger under dual-task conditions (6.4% average, Experiments 1–3) than under single-task conditions (2.5% average, Experiments 1–3). Thus, divided attention decreased selectivity. Furthermore, the results across several experiments provide initial answers to the three questions regarding the source of congruency effects that were raised in the introduction. First, because the stimuli were identical across single- and dual-task conditions, the differential congruency effects indicate that selection played a role rather than it being an effect due entirely to some form of stimulus-driven interactive processing. Second, the congruency effect for dual tasks persisted even when the stimuli were presented sequentially (Experiment 2). This is consistent with the locus of the effect being in later processes rather than in immediate stimulus-driving processes. Third, the congruency effect for dual tasks disappeared with high-contrast stimuli (Experiment 4). This is consistent with models that attribute the effects to a graded process rather than to an all-or-none process. Appendix B provides formal examples of each of these types of process—the weighting model (graded) and the substitution model (all or none).

#### Dual-task deficits

In addition to the main results that were the focus of the study, the first three experiments all found small, barely detectable dual-task deficits of around 2%. Such small deficits are consistent with the previous experiments of Bonnel et al. (1992) and Graham et al. (1985). They contradict the claims of some studies (e.g., Lee et al., 1999; Pastukhov et al., 2009) that all tasks have similar effects of divided attention. One reason for the apparent differences between studies might be the use of a fixed order of responses versus an unpredictable order of responses. With a fixed order, one can start to prepare for the first response rather than maintaining both decisions. This will make different tasks more homogeneous (see also Lyphout-Spitz et al., 2024). We suggest that using an unpredictable order provides better insight into the diverse effects of divided attention.

### Generality of main results

The current experiments, which used widely separated stimuli, showed congruency effects that were larger for dual tasks than single task. How general is this result? Our lab has conducted similar dual-task studies using a variety of tasks and stimuli and the results have been of several sorts. In four experiments using judgments of masked words, a similar pattern of differential congruency effects for single and dual tasks occurred (average congruency effect of 6% for dual tasks and 3% for single tasks; White et al., 2018, 2020). Other experiments yielded smaller congruency effects overall, and they did not differ across single- and dual-task conditions. Two experiments using judgments of masked objects (rather than words) showed this pattern (average congruency effect of 2% for dual tasks and 2% for single tasks; Popovkina et al., 2021). Three experiments using judgments of words that were presented in simultaneous noise, rather than being backward masked, showed this pattern (average congruency effects of 2% for dual tasks and 2% for single tasks; Palmer et al., 2020). Finally, there has also been one exception to these two patterns. In an experiment using color discrimination of masked stimuli (colored words), relatively large congruency effects were found with both single and dual tasks (average congruency effects of 6% for dual tasks and 6% for single tasks; White et al., 2020). In summary, we mostly find two patterns:

- Larger congruency effects in dual tasks than single tasks – this was found for detection (no mask) tasks and for masked-word judgment tasks.
- Non-differential and small congruency effects – this was found for masked- object-judgment tasks and word-in-noise judgment tasks.

There are hints in the literature for why the results for detection and tasks with masked stimuli might differ from other tasks. When the task is detection, something like what Bacon and Egeth (1994) described as a singleton-detection strategy can be adopted which increases sensitivity for detection by minimizing selectivity. Reducing selectivity, however, also increases susceptibility to congruency effects. When the task involved mask, Morgan et al. (1998) found that masking increased effects of divided attention and decreased the effectiveness of spatial selectivity. Again, decreased selectivity increases susceptibility to congruency effects. To conclude, the pattern of congruency effects varies with the details of the task and stimuli.

### Relation to the flanker paradigm

This article is focused on the observation of larger congruency effects in dual tasks compared to single tasks in the spatial filtering paradigm which, as elaborated in the introduction, is well suited for isolating spatial selection processes. Similar findings, however, have been reported from studies using the flanker paradigm, which involves processes beyond purely spatial selection.

Hubner and Lehle (2007) conducted several experiments combining the flanker paradigm with a speeded dual task. A target digit was presented at fixation with two flanker digits to either side. These flankers were always identical to one another and were displayed until the response(s) were made. The required judgment was to indicate the parity (odd vs. even) of the target using one of two keypresses. For example, a congruent case would be a “4” target surrounded by two “6” flankers; and, an incongruent case would be a “4” target surrounded by two “7” flankers. The innovation was to add a dual-task condition with a second parity judgment of the flankers. The second judgment was made using a different hand and always followed the first response. They also varied the onset of the flankers from being simultaneous with the target to following the target by several hundred milliseconds. Thus, these speeded dual-task conditions had the typical features of the psychological refractory paradigm (cf. Pashler, 1994).

There were several experiments establishing the generality of their results, but here we focus on Experiment 1 and on the conditions with simultaneous targets and flankers. First consider the response to the targets. For single-task blocks, the flanker effect was 20 ms (~ 475 vs. ~ 495 for congruent and incongruent, respectively), whereas for dual-task blocks, the flanker effect was 180 ms (~ 760 vs. ~ 940 ms). Accuracy was high in all conditions with a mean of 3% errors and there were no significant differences across congruency conditions or between single and dual tasks. Thus, the congruency effect on response time was almost an order of magnitude larger for the dual task than the single task. This result was replicated and generalized in multiple experiments in this paper and in a following paper (Lehle & Hubner, 2009). In particular, they showed how these effects were subject to strategy. For example, the larger congruency effect with dual tasks was reduced by mixing the single and dual-task trials but it did not go away. There is little doubt that congruency effects in the flanker task are increased in the context of a dual task. Further studies of flanker-congruency effects in the dual-task context (but without the single task comparison) can be found in Rieger and Miller (2020). In summary, the increase in congruency effects with a dual task appears to be general to both spatial filtering, which reflects purely spatial selection, and flanker paradigms, which reflect other forms of selection and probably interactive processing as well.

#### Interpretation of results

As discussed above, the fact that identical stimuli and tasks were used in the single- and dual-task conditions of the current experiments, rules out a purely stimulus-driven interactive process account of the congruency effects, and confirms some role for the selection process. A pure selection account, however, also has problems accounting for the larger body of evidence. A pure selection account for the current experiments requires a graded selection process such as a contrast gain mechanism. Yet previous experiments that used a spatial-filtering task similar to that of the current study, but with small separations, ruled out a graded selection process, and instead was consistent with an all-or-none selection process (e.g., Yigit-Elliot et al., 2011). Given the similarity of the experiments, it would be ad hoc to propose that selection is all-or-none for some spatial filtering tasks and graded for others.

**Fig. 7:**
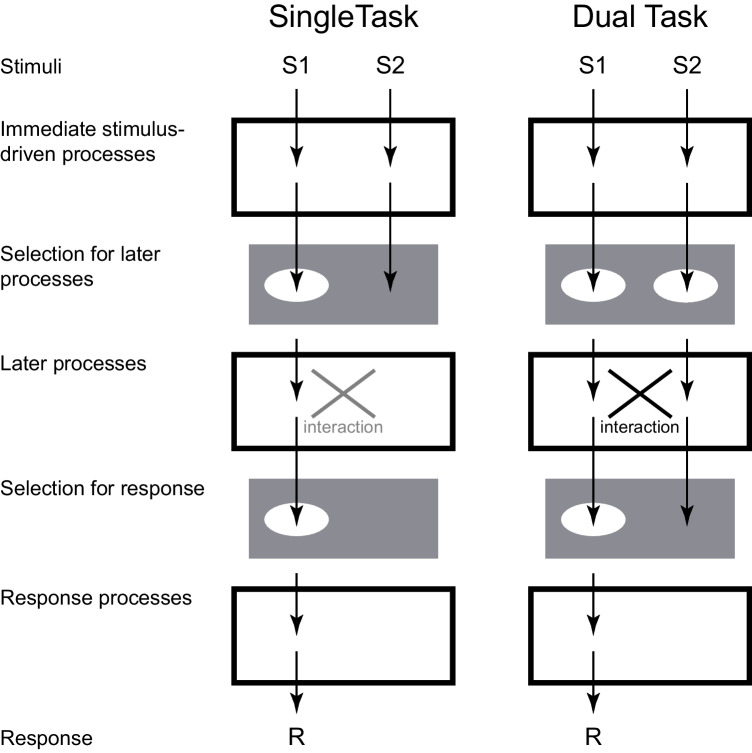
An illustration of the proposed processing sequence of our working hypothesis. The processing sequence for each stimulus goes from top to bottom. The processes for a single task and a dual task are shown in the left and right columns, respectively. Interactions occur only in the later processes and only for a dual task. For this illustration, stimulus S1 is relevant to the specific response

Our working hypothesis, which is illustrated in Fig. 7, is that both selection and interactive processes contributed to congruency effects in the current experiments. Specifically, we propose a *two-process hypothesis* according to which selection is all-or-none, as concluded in Palmer and Moore (2009), but in addition, a graded interactive process in later processes can impact performance when multiple representations must be maintained. In dual-task conditions, two representations must be maintained and final selection between them occurs at the time of decision for each response. In the single-task conditions, only one representation must be maintained. Under our working hypothesis, representations are subject to interference through interactive processing in some later process, for example, in memory (Oberauer & Lin, 2017), or during decision (Hommel, 1998; Logan & Gordon, 2001). If two noisy representations are maintained in the dual-task conditions, this interactive processing gives rise to congruency effects (Experiments 1–3). When the two representations are less noisy, however, as in the case of high-contrast stimuli, interactive processing has little to no impact on performance (Experiment 4). If there is only one representation being maintained, as is the case in single-task conditions, interactive processing is not relevant and does not impact performance. It is in this case that the earlier all-or-none selection prevents an effect of the later graded selection.

The two-process hypothesis has two positive properties. First, it is parsimonious in that only a single all-or-none selection process is proposed and the potential for impact from interactive processing is the same for both single and dual-tasks, it just is not relevant for single tasks in which only a single representation must be maintained and there is no other representation to interact with. Second, it maintains the understanding of interactive processes as being non-selective which is typical of such accounts (e.g., Eriksen & Schultz, 1979; Hommel, 1998; Navon & Miller, 1987; Oberauer & Lin, 2017).

#### Alternative theories

Perhaps the most relevant alternative theory to our two-process hypothesis is an account of congruency effects that was proposed by Logan and Gordon (2001). This study is particularly relevant to the current study because it used spatial filtering rather than the flanker paradigm. They proposed that congruency effects derive from a graded interactive process (crosstalk) within a late process. In addition, they proposed that the difference in the size of congruency effects in dual- versus single-task conditions derives from a top-down control mechanism (the $\beta$ parameter in their model) that modulates the degree of crosstalk. In the terms that we have been using, their model includes a graded selection mechanism that is more selective with single tasks than dual tasks. An advantage of the two-process hypothesis that we have proposed is that all-or-none selection accounts not only for the difference in congruency effects in dual- versus single-task conditions, but also the evidence of all-or-none selection with small separations (Palmer & Moore, 2009; Yigit-Elliot, 2012; Yigit-Elliot et al., 2011).

Another alternative theory was described by Hubner and colleagues across several papers (Hubner et al., 2010; Lehle & Hubner, 2009). It was developed in the context a flanker task that was generalized to include elements of the PRP paradigm as discussed above. The theory has both an early selection mechanism, which is subject to inputs from irrelevant stimuli, and a later selection mechanism that is not (Hubner et al., 2010). In addition, as presented in Lehle and Hubner (2009) it includes a version of central capacity theory (Tombu & Joicoeur, 2003) that assumes incongruent stimuli in a dual task must be resolved by control processes that consume part of central capacity. This provides an account for larger congruency effects in dual-task conditions relative to a single task. Again, an advantage of the two-process hypothesis that we have proposed is that the all-or-none selection process accounts for both the difference in congruency effects in dual- versus single-task conditions and all-or-none selection with small separations (Palmer & Moore, 2009; Yigit-Elliot, 2012; Yigit-Elliot et al., 2011).

In summary, our two-process hypothesis and the two theories just reviewed, all have different domains and different strengths. We focus on how each theory accounts for the difference in congruency effects for single and dual tasks. Logan and Gordon (2001) do this directly by modulating a graded selection process. Huber and colleagues do it by adding an additional claim to central capacity. Our working hypothesis does it as a side effect of an all-or-none selection process. While we think our working hypothesis is the simplest, it will take integrative studies combining the relevant phenomena to fully discriminate these possibilities.

## Conclusion

Using widely separated stimuli in a spatial filtering paradigm, we consistently found an effect of congruency under dual-task conditions, but little to no effect of congruency for single-task conditions. Because stimuli were identical in single-task and dual-task conditions, this indicates that spatial selectivity was reduced for dual tasks, rather than the difference being due a pure stimulus-driven interactive process. In addition, the dual-task congruency effect persisted with sequential stimulus presentation indicating that the locus of the effect is in a later process (e.g., decision), rather than an early immediate process (e.g., perception). Finally, the dual-task congruency effect disappeared with high-contrast stimuli, indicating that the effect was due to some kind of graded process such as attenuation rather than to an all-or-none process such as blocking. Our working hypothesis is that there is an all-or-none selection process, which can also account for previous results with close stimulus separations (Palmer & Moore, 2009; Yigit-Elliot, 2012; Yigit-Elliot et al., 2011) and an interactive process that is graded in nature and occurs late in processing. The all-or-none selection can protect against interactive processing in a single task but not in a dual task when representations of both stimuli must be held in memory.

## Figures and Tables

**Fig. 8:**
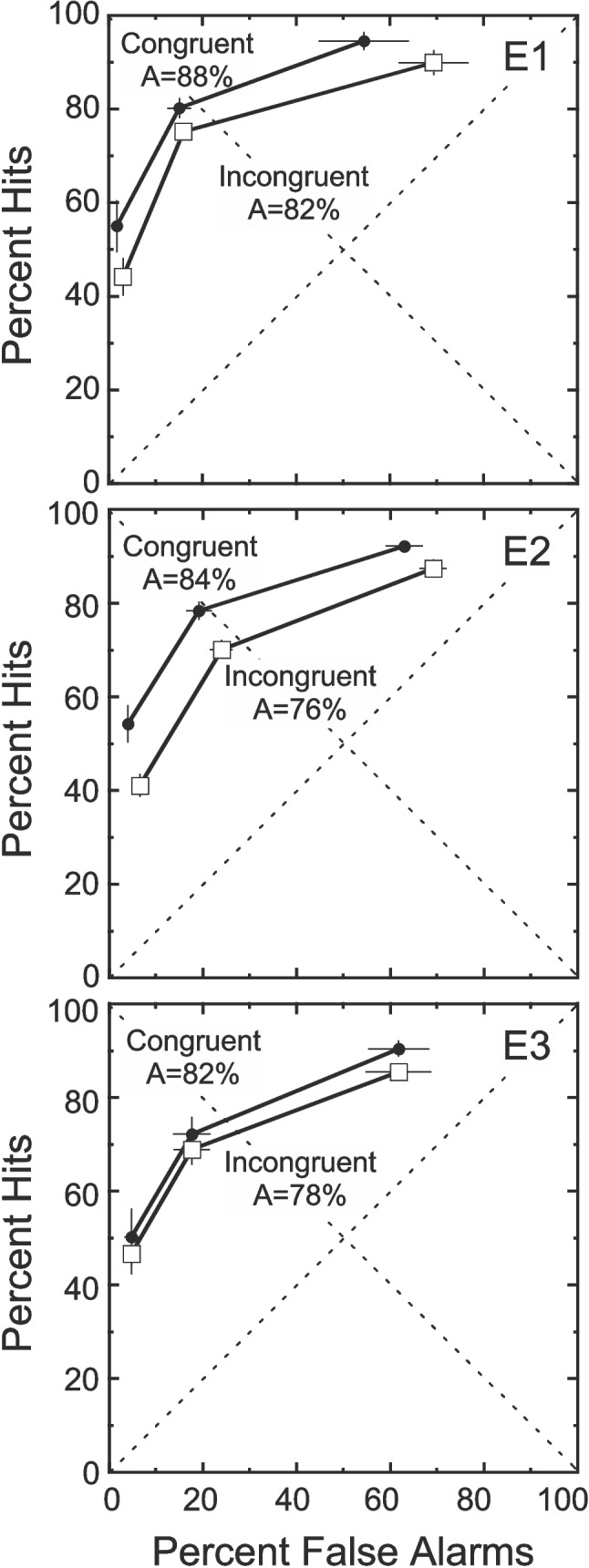
ROC functions for congruency effects. Each panel contains an ROC plot in which the percent hits is plotted against the percent false alarms. Congruent conditions are shown by the solid symbols and incongruent conditions are shown by the open symbols. These ROC functions correspond well with the analysis using estimates of the area under the ROC function

**Fig. 9:**
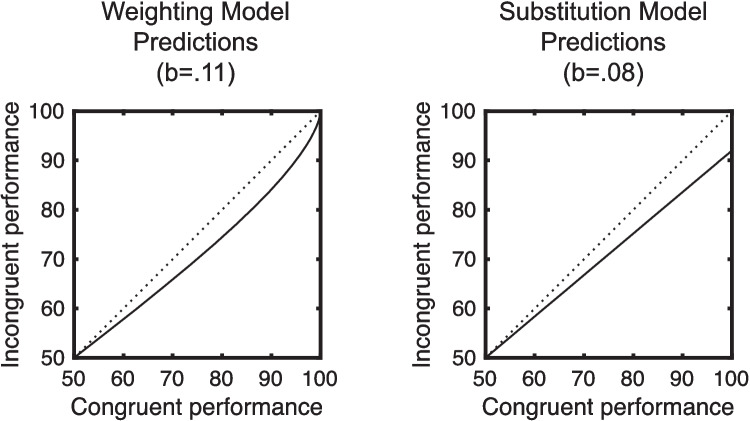
Congruency effects for weighting and substitution models. For both, the percent correct performance in the incongruent condition is plotted against the percent correct performance in the congruent condition. For the weighting model in the left panel, the predicted congruency effects disappear with high discriminability. For the substitution model in the right panel, the predicted congruency effects persist even with high discriminability. The results of the current experiments are qualitatively consistent with the weighting model

**Fig. 10:**
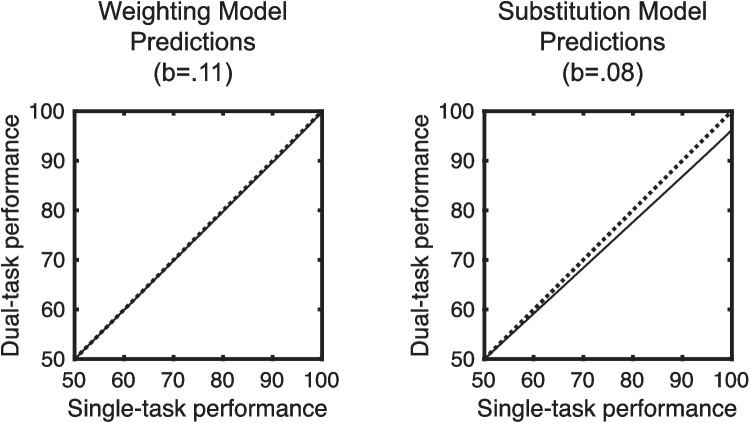
Dual-task deficits for weighting and substitution models. For both, the percent correct performance in the dual-task condition is plotted against the percent correct performance in the single-task condition. For the weighting model in the left panel, there are essentially no dual-task deficits. For the substitution model in the right panel, there are dual-task deficits that arise from selection errors or interactive processing rather than from capacity limits

**Fig. 11:**
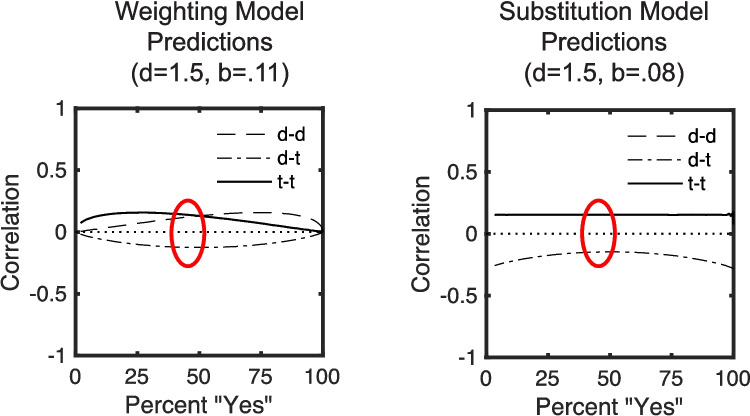
Predicted correlations for the weighting and substitution models. For both, the predicted correlation is plotted against the percent of “yes” responses. The three curves are for trials with two targets (t-t), a distractor and a target (d-t), and two distractors (d-d). The predictions for p(“yes”) = 0.45 are circled for emphasis because these conditions match the current experiments. For the weighting model in the left panel, positive correlations are predicted for target-target and distractor-distractor trials and negative correlations for the distractor-target trials. For the substitution model in the right panel, identical positive correlations are predicted for target-target and distractor-distractor trials, and negative correlations for the distractor-target trials. The results of neither model are qualitatively consistent with the current experiments

## Data Availability

The data are available in a repository of the Open Science Framework: osf.io/2zgdh/

## Appendix A

### Analysis of the rating data

In the body of this article, we summarized performance by *A*_*ROC*_: the percent area under the ROC curve. As discussed in the *Methods*, this measure is an estimate of the unbiased percent correct. In this appendix, the rating data is described from which *A*_*ROC*_ is estimated, and the congruency effects are broken down by targets versus distractors.

Rating data such as used here can be summarized by an ROC graph that plots the percent hits against the percent false alarms. Figure 8 shows such parametric plots for the congruency effects in each of the three main experiments. Such graphs represent the cumulative percent of responding “yes” for each consecutive rating (rating 1, rating 2 or less, rating 3 or less). There is no point for the fourth rating because one must use one of the 4 ratings so the result always falls at the point (100, 100). If performance is at chance, the points fall on the positive diagonal; if performance is perfect, it falls in the upper left corner with 100% hits and 0% false alarms. Finally, if performance for a given rating is unbiased (probability of a “yes” is 0.5), then that point falls on the negative diagonal.

For all experiments, the three points on the ROC curve formed a negatively accelerated function that is typical of predictions from signal detection theory based upon comparing random variables to a decision criterion (Green & Swets, 1966). The ROCs clearly deviates from a linear function that is predicted by the high threshold theory (a line from (0, *x*) to (100, 100) with *x* between 0 and 100) in which one guesses when not detecting the target. Thus, one can rule out a simple version of the high threshold model for these experiments.

The effect of congruency on the ROC is illustrated in Fig. 8. Each panel shows the results of the dual-task condition broken down by congruent and incongruent trials. Only the dual-task conditions are shown because the congruency effect is larger for that condition than the single-task condition. The solid circles show performance for the congruent conditions and the open squares show performance for the incongruent condition. The congruency effects were consistently significant as reported in the body of the article. Here one can also see the ROCs were shifted between the congruent and incongruent conditions as expected for a change in sensitivity.

We can further break down the congruency effect into its components. For hits, the congruent target – target pair can be compared with an incongruent target – distractor pair. For correct rejections, the congruent distractor – distractor pair can be compared to the incongruent distractor– target pair. For targets, the effect of congruency on hits was 5 ± 1, 8 ± 1, and 3 ± 3 for Experiments 1, 2, and 3, respectively. For distractors, the effect of congruency on correct rejections was 1 ± 1, 5 ± 3, and 0 ± 1 for Experiments 1, 2, and 3, respectively. For individual experiments, these results were not significant. Instead, experiments were combined as if all the subjects were in one big experiment. The combined congruency effects for targets was a significant 6.7 ± 1.3% (95% CI 4.0, 9.4, *t*(17) = 5.22, *p* < 0.001, two tailed). The combined congruency effects for distractors was a not significant 3.1 ± 1.6% (95% CI - 0.3, 6.4, *t*(17) = 1.92, *p* = 0.071, two tailed). The difference is a significant 3.6 ± 1.7% (95% CI 0.1, 7.1, *t*(17) = 2.18, *p* = 0.043, two tailed). In summary, this pattern is consistent with the congruency effect being larger for hits (two targets) relative to correct rejections (two distractors).

In our previous studies, we did not find consistent differences in congruency effects for target and distractors. In White et al. (2020), there were similar congruency effects for both hits and correct rejections. In Popovkina et al. (2021), there were what is called a *two-target effect* (Duncan, 1980): worse performance for hits with two targets (a negative congruency effect). Thus, this detailed pattern of effects seems to vary with the task and stimulus. Perhaps the pattern found in this article is specific to detection experiments.

## Appendix B

This appendix describes two models for congruency effects. One depends on a graded process such as selection by attenuation or crosstalk, and the other by an all-or-none process such as selection by blocking or substitution. They are intended as specific examples of the general theories proposed in this article.

### Definition of the standard unlimited-capacity, parallel model

For our basic model, assume an unlimited-capacity, parallel process. Denote the two tasks: *i* = 1, 2. Assume the relevant evidence from each stimulus for each task correspond to random variables *S*_*1*_ and *S*_*2*_. Further assume these random variables are independent and identically distributed, and have unit variance. With these strong independence assumptions, this is often referred to as the *standard* version of this model. For the tasks considered here, stimuli are either targets or distractors: the targets were Gabor patterns in noise and the distractors were noise alone. Assume the difference between target and distractor representations corresponds to a shift in the mean of their random variables: *S*_*target*_ = *S*_*distractor*_ + *d*. The *sensitivity* parameter *d* is in units of the standard deviation of these random variables.

The *criterion* parameter *c* allows one to adjust the response bias for the decision. It is also in units of the standard deviation of the random variables. Following signal detection theory, assume the decision rule for task *i* is:

If *S*_*i*_ > *c*, then respond “yes”, otherwise, “no”.

Following this rule, the proportion of “yes” responses for task *i* is:

$$P_i\left(^{\prime \prime} yes^{\prime \prime}\right)=P\left(S_i>c\right).$$

In fact, for the rating task one actually needs three criteria to generate 4 possible ratings, but we ignore that modest complication in this appendix. In summary, this standard unlimited-capacity, parallel model has two parameters: *d* is the sensitivity parameter, and *c* is the decision criterion.

### Alternative models for congruency effects

In the following section, we present two models of congruency effects built upon the just described model of unlimited-capacity, parallel processing. One is a graded model and the other is an all-or-none model. These models can be interpreted as models of either selection errors or interactive processes; and, they can have a locus in either immediate or later processing.

#### A weighting model of congruency effects

In this section, we define a graded model of congruency effects. This weighting model can be interpreted as a selection model with graded weights for decision (Kinchla & Collyer, 1974). Alternatively, this model can be used to describe interactive processing such a crosstalk (Navon & Miller, 1987). The equations do not distinguish these concepts. To distinguish them, one must assume something about the effects of other manipulations such as cueing single-versus-dual tasks or the simultaneous-versus-sequential displays.

Assume all of the features of the previously described unlimited-capacity, parallel model concerning stimulus representation, target representation, the criterion, and the decision rule. The new feature is to revise the decision variables *S*_*1*_ and *S*_*2*_. Denote a new decision variable for each task as *S'*_*1*_ and *S'*_*2*_. Assume these are a function of both of the stimulus representations *S*_*1*_ and *S*_*2*_:

$${{S}^{\prime}}_{i}=\left(1-{b}_{weight}\right){S}_{i}+{b}_{weight}{S}_{j},where\; j\ne i.$$

The parameter *b*_*weight*_ is a weight for the inclusion of information from the irrelevant stimulus representation. It is zero for the case of no weight for irrelevant information and is 0.5 for equal weight. This form of the equation fixes the variability of the decision variable to 1. Since both random variables begin with a unit variance, the weighting by *b*_*weight*_ and (1-*b*_*weight*_) maintain the variance at 1. Using this addition, the final proportion of “yes” responses is thus given by:

$${P}_{i}\left(^{\prime \prime}yes^{\prime \prime}\right)=P\left({{S}^{\prime}}_{i}>c\right).$$

#### A substitution model of congruency effects

A substitution model is appropriate when selection fails in an all-or-none way (e.g., Palmer & Moore, 2009) or when an interactive process substitutes one stimulus representation for another (e.g., Ester, et al., 2014). This model is also often called a “mixture model” because performance on an average of trials is a mixture of trials with or without the substitution. As before, assume all of the features of the unlimited-capacity, parallel model. The new feature is that on some trials, the representation of the relevant stimulus is replaced by the representation of the irrelevant stimulus. The stimuli are represented by random variables *S*_*1*_ and *S*_*2*_ for the two tasks. Suppose the relevant task (and stimulus) is *i*, the probability of a “yes” response in this task is a mixture of trials in which the decision was based on the relevant stimulus and trials in which it was based on the irrelevant stimulus:

$${P}_{i}\left(^{\prime \prime}yes^{\prime \prime}\right)=\left(1-{b}_{sub}\right)P\left({S}_{i}>c\right)+{b}_{sub}P\left({S}_{j}>c\right), j\ne i.$$

The parameter *b*_*sub*_ is the probability of basing the decision on the irrelevant stimulus. This parameter can be interpreted as either the probability of a selection error in a model of selection, or as the probability of a substitution in a model of interactive processes.

### Comparing predictions of weighting and substitution models

#### Congruency effects

Next consider the predictions for congruency effects. Begin by considering the weighting model assuming moderate amounts of selection error/crosstalk (*b*_*weight*_ = 0.11). This value of *b*_*weight*_ was chosen to match the 6% congruency effects observed here. To generate specific predictions, assume equal-variance Gaussian distributions for targets and distractors.

The left panel of Fig. 9 shows the congruency effects predicted by the weighting model. In it, incongruent performance is plotted against congruent performance. A congruency effect is shown by a downward deviation from the dotted identity line. For *b*_*weight*_ = 0.11, the congruency effect is about 6% when the mean performance is 85% correct. As discriminability improves toward perfect, the congruency effect falls back to zero.

Next consider the predictions of a substitution model with selection or substitutions of irrelevant stimuli rather than weighting. Again, assume unlimited capacity, a moderate amount of selection error/substitution (*b*_*sub*_ = *0.08*), and equal-variance Gaussian distributions. As before, this value of *b*_*sub*_ was chosen to match the 6% congruency effects observed here.

The right panel of Fig. 9 shows the congruency effects predicted by the substitution model. The new feature of the prediction is that the congruency effect grows proportionally with performance. For *b*_*sub*_ = 0.08, the congruency effect is about 6% when the mean performance is 85% correct. As discriminability improves toward perfect, the congruency effect grows to a maximum of 8%. The value of this maximum congruency effect is equal to the selection parameter *b*_*sub*_. In Experiment 4, performance for high contrast stimuli approached perfect and the congruency effect disappeared. This is consistent with the weighting model and not the substitution model.

#### Dual-task deficits

Next consider the dual-task deficits predicted by these two models. The predictions of the weighting model with *b*_*weight*_ = 0.11 are shown in the left panel of Fig. 10. The dual-task deficit is very small and is not visible in this figure. The deficit is about 0.3% for a single-task performance of 85% correct. Thus, adding weighted selection/crosstalk causes little change to the dual-task deficit for the two-choice tasks considered here.

The right panel of Fig. 10 shows the dual-task deficits for the substitution model with *b*_*sub*_ = 0.08. Unlike the previous model, adding selection error/substitutions does change the dual-task deficits. There is now a dual-task deficit even when there is unlimited capacity. For a single-task performance of 85% correct, the dual-task deficit is about 3%. This is because substitution hurts performance in the incongruent condition and cannot help performance in the congruent condition. Thus, a dual-task deficit is a necessary prediction of selection by substitution. The absence of a dual-task deficit is by itself evidence against substitution.

Neither of these predictions match the observed results of a 2% deficit at a single-task performance of 85% correct. The weighting model does not predict any deficit and the substitution model for a 6% congruency effect predicts a deficit of half that size: 3%. A bit too large. One way to get closer to the observed results is the modify the weighting model to be asymmetric. If the advantage of the congruent condition is smaller than the disadvantage of the incongruent condition, that causes a dual-task deficit. Such an asymmetric weighting model is also consistent with the results of Experiment 1 in which performance for the single-stimulus condition is closer to performance in the congruent condition relative to the incongruent condition.

#### Response correlations

Next compare the response correlations predicted by the two models. Predictions of the weighting model are shown in the left panel of Fig. 11 as a function of the probability of a “yes” response. These predictions are for a dual-task condition with *d* = *1.5* and *b*_*weight*_ = *0.11*. This intermediate discriminable condition roughly matches the current experiments. There are positive correlations predicted for the target-target and distractor-distractor trials and negative correlations predicted for the target-distractor trials. The correlation predictions for the case with probability of “yes” equal to 0.45 are highlighted with a red circle because these are the conditions observed in the experiments. The correlations are strongly dependent on the weight parameter *b*.

The predicted correlations of the substitution model are in the right panel. These predictions are for *d* = 1.5 and *b*_*sub*_ = 0.08 which roughly match the conditions of the current experiments. There are positive correlations for the target-target and the distractor-distractor trials (hidden under the target-target trials). And there are negative correlations for the target-distractor trials. These results are qualitatively similar to the weighting model.

Neither of the predicted patterns match the patterns observed for the current experiments. In the experiments, the correlation was positive for only distractor-distractor trials (see also the color task of White et al., 2020). That pattern can be obtained by changing the weighting model so that there are separate terms for target and distractor conditions (not shown). A model with weights driven by noise in the distractor conditions alone predicts that the positive correlations occur primarily for distractor-distractor trials. Think of this modification as introducing correlated noise for the distractors. Thus, this noise-specific weighting model results in a pattern of correlation more like that found in the current experiments.

#### Summary

The predictions of the weighting and substitution models differ in clear cut ways. The congruency effect is predicted for highly visible stimuli for the substitution model but not for the weighting model. And, dual-task deficits always occur for the substitution model but need not occur for the weighting model. In the current study, the observed results favor the weighting model but it needs elaboration to capture all of the current results.

### Computational methods

All predictions were based on simulation. Our goal in these simulations was to achieve at least three digits of accuracy. We confirmed the precision of our estimate by making multiple estimates and testing that the standard deviation of that sample was smaller than 1/1000 of the estimated value. To achieve this goal, 1,000,000 trials were simulated for each predicted proportion. To estimate the response correlations, 10,000,000 pairs of responses were simulated.

To estimate the area under the ROC curve (*A*_*ROC*_), we numerically integrated a simulated ROC curve. Care was necessary because the area is underestimated if one uses steps that are too large or limits that are not extreme enough. The curve was sampled by varying the criterion in steps of 0.005 and extending the limits of integration to achieve proportions of hits and false alarms that differed from 0 or 1 by less than 0.005. These choices were based on the testing with a range of yet smaller values and picking the step size that yields area estimates that were within 1/1000 of the estimate made with much smaller steps and more extreme limits.

## Code availability

Software is available upon request to John Palmer at jpalmer@uw.edu.
